# The role of concentration in electrolyte solutions for non-aqueous lithium-based batteries

**DOI:** 10.1038/s41467-022-32794-z

**Published:** 2022-09-06

**Authors:** Guinevere A. Giffin

**Affiliations:** 1 Fraunhofer R&D Center Electromobility, Fraunhofer Institute for Silicate Research, Neunerplatz 2, 97082 Würzburg, Germany; 2 Chair of Chemical Technology of Materials Synthesis, Julius-Maximilians-University Würzburg, Röntgenring 11, 97070 Würzburg, Germany

**Keywords:** Batteries, Energy, Materials science, Batteries, Energy storage

## Abstract

The quest for high-energy electrochemical energy storage systems has driven researchers to look toward highly concentrated electrolytes. Here, the author discusses the recent progress and future perspectives of such electrolytes and their ability to improve the performances of lithium-based batteries.

## Teaser

The main components and, most notably, the concentration of the non-aqueous electrolyte solution have not significantly changed since the commercialization of Li-ion batteries in the early 1990s. However, the quest for electrochemical energy storage systems with high-energy content has driven researchers to reconsider the suitability of the “standard” one molar concentration and look toward highly concentrated electrolyte solutions. However, the interplay between the fundamental electrolyte properties and the cell performance is not consistent with what would be expected based only on the electrolyte ionic conductivity. Here, the recent progress and future perspectives on the correlation between the physicochemical properties of non-standard electrolyte solutions and their ability to improve the energy storage performances of lithium-based batteries are discussed.

## Main

Since Sony’s commercialization in 1991^1^, numerous advances in non-aqueous lithium-ion batteries have led to many products^1,2^. Efforts to enhance the energy density and specific energy have resulted in decades of intensive research to improve the electrode active materials. Consequently, electrode active material development has far outpaced the advances in electrolyte chemistry. The composition of the electrolyte solution, despite its critical role in the cell as the ionic conductor to transport lithium ions between the electrodes, is basically the same today as in the early 1990s^1,2^. A “standard” electrolyte formulation contains a mixture of linear and cyclic carbonate solvents as a 1 molar (M) salt solution, where the salt is typically lithium hexafluorophosphate (LiPF_6_). This “standard” electrolyte is then tailored to the specific cell chemistry primarily through variations of the carbonate solvent and incorporation of proprietary mixtures of additives^3^. These additives, which may include solvents, salts, or other molecules which would not be considered solvents, are generally used in smalls amounts in comparison to the amount of electrolyte solvent (in the review by Xu arbitrarily set at 10%, no indication of wt% or vol% given; furthermore, amounts above 10% are considered co-solvents)^4^. More recently, Solchenbach et al. suggested that the ratio of the additive to the active material may be more relevant than a particular concentration^5^. However, the ideal amount of any particular additive likely depends on its function in the cell and the amount needed to obtain the desired effect without having a significant negative influence on other properties impacting the performance. Additives fulfill many purposes in the cell, such as film formers (i.e., sacrificial additives) for the formation of solid electrolyte interphase (SEI), cathode electrode interphase (CEI) or compounds to enhance the safety of the system (e.g., flame retardants)^3^. As an example, the amount of a CEI additive is likely to be lower to avoid increasing the interphase resistance than, e.g., the amount of a flame retardant, where the higher contents are needed to influence the self-extinguishing time.

**Fig. 1:**
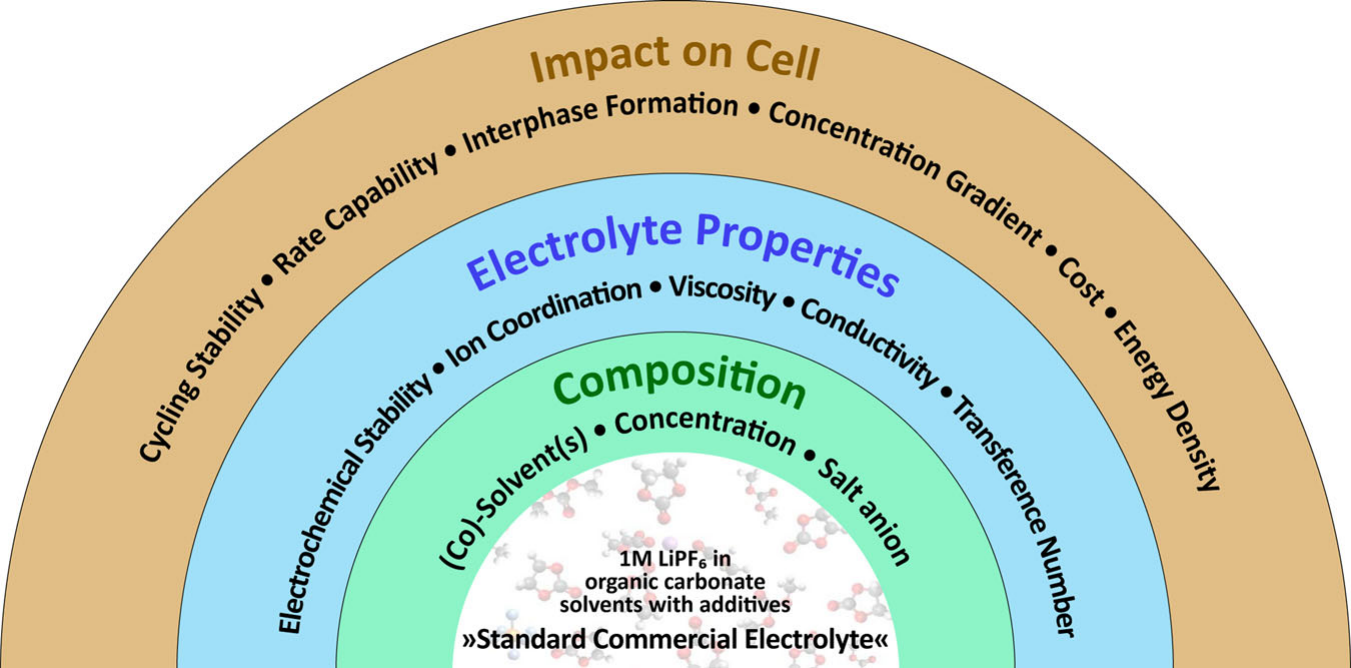
The electrolyte playing field. In the middle of the figure, the standard commercial lithium-ion battery electrolyte solution is described. This standard electrolyte can be changed (green ring) not only by varying the composition (e.g., the solvents or the salt anion) but also by changing the concentration. Changes in the composition affect the electrolyte properties (blue ring), which subsequently impact the cell performance (orange ring).

In terms of the main electrolyte solution components, there remains a rather large playing field that has only started to be explored in recent years (Fig. 1). For example, the concept of ethylene carbonate (EC)-free electrolytes has been explored to enhance high-voltage operation^6^. The aspect of salt concentration is a particularly interesting aspect to be explored. Changes in the concentration directly affect the solvation of the Li^+^ ions in the solution and subsequently all of the other electrolyte properties, including the formation of the interphases (SEI and CEI). This commentary article will focus specifically on highly concentrated electrolyte solutions (also known as solvent-in-salt systems^7^) and address aspects relating to future electrolyte development and their implementation in lithium-based batteries.

Adequate lithium-ion transport properties are necessary to satisfactorily guarantee electrochemical energy storage performances. Conventional wisdom (i.e., the understanding and explanation of electrolyte properties generally accepted by experts in the field of battery electrolyte solutions) says that this is achieved through a high conductivity and low viscosity electrolyte. In most non-aqueous lithium-ion conducting electrolyte solutions, the maximum bulk conductivity occurs at an approximately 1 M salt concentration. It is, therefore, no coincidence that the “standard” electrolyte concentration is 1 M. Borodin et al. have called this the “1 molar (M) legacy”^8^. This maximum conductivity results from a trade-off between the number of charge carriers and the ionic mobility of these charge carriers. The number of charge carriers is determined by the salt dissociation, while the ionic mobility is primarily associated with the viscosity of the electrolyte media. In the 1 M concentration regime, the lithium ions are solvated by the “conventionally-used” carbonate solvent(s), and the anions are largely considered to be “free” (often referred to as solvent-separated ion pairs or SSIP)^9^. The structure of the solvation shell, i.e., the lithium ions and the directly-coordinated electrolyte components, depends on both the nature of the solvent and the salt anion. Furthermore, there is a significant amount of uncoordinated solvent, i.e., solvent molecules beyond the first solvation shell. At high salt concentrations (a definition of “high concentration” is discussed in the next paragraph), the lithium-ion coordination is very different. The lithium ions are coordinated by both the anions and the solvent molecules. Moreover, there are few, if any, free solvent molecules that influence not only the transport properties of the electrolyte but also the interaction between the electrolyte and the other components in the cell, e.g., in SEI/CEI formation.

The comparison of the “1 M” and “high-salt concentration” electrolyte solutions leads to the question, what is “high concentration”? Unfortunately, there is no single answer to this question as the boundaries between different concentration regimes of non-aqueous battery electrolyte solutions highly depend on the definition criteria. From the perspective of ideality, from which almost all of the classical equations to describe electrolyte behavior were derived, all of the ions in a solution must be completely dissociated, participate in diffusion and migration, and move independently (i.e., are not influenced by other ions)^10^. However, battery electrolyte solutions deviate from ideality already at concentrations below 0.1 M^11^; thus, even the “standard” 1 M electrolyte solutions can be considered concentrated electrolytes. Therefore, a practical definition of a “concentrated electrolyte” differs significantly from the ideal definition based on independent ion movement. In a possible effort to reconcile these differences, the term “super-concentrated” has appeared in the literature and has been loosely associated with concentrations above approximately 3 M in aprotic solvents^8^.

It has been recently suggested that one way to classify practical electrolytes (i.e., electrolytes used in technologically-relevant electrochemical energy storage devices) into concentration regimes may be based on the nature of the ion-solvation shell^8,10^. As noted above, in highly-concentrated electrolytes (or “super-concentrated” electrolytes), there are few, if any, free solvent molecules and the anions are present in the first solvation shell. In contrast, in “low concentration” electrolytes (i.e., less than 3 M based on the molarity definition noted above), there are solvent molecules which are not directly coordinated to cations in solution and, thus, are free.

The concentrations above are given in molarity (i.e., moles of solute per liter of solution), as is often used in the literature. However, molarity is not necessarily the best measure of concentration for highly-concentrated electrolyte solutions as the density changes significantly with the concentration. As an example, electrolytes containing lithium bis(fluorosulfonyl)imide (LiFSI) in EC increased in density from 1.38 g cm^−3^ at 0.63 M to 1.71 g cm^−3^ at 5.67 M^12^. Therefore, other units to express concentration, such as molality (i.e., moles of solute per 1 kg of solvent) or mole ratio (moles solute:moles solvent), are better suited, particularly when comparing electrolytes over a wide concentration range.

As an extension of the conventional wisdom referred to above, poorer cell performance is expected with high-concentration electrolytes when bulk conductivity is lower and the viscosity increases. This assumption is largely derived from the picture at lower concentrations where a vehicular transport mechanism (i.e., where the lithium ions move through the electrolyte with its solvation shell) makes a substantial contribution to the lithium-ion conductivity^8^. In this case, “lower concentration” depends on the solvent and may be considered to be up to approximately 3 M for carbonates^8^. Examining the results from research studies on highly-concentrated electrolyte solutions leads to questions about this conventional wisdom and, thus, the usefulness of the (bulk) electrolyte conductivity as the key evaluation criterion. Can other transport mechanisms, such as structural diffusion, where the lithium-ion is transported through the exchange of the components in the first solvation shell (i.e., ion association-dissociation processes)^8^, also effectively transport “sufficient” lithium to the electrode? Can a high concentration of lithium ions minimize the effects of concentration gradients in the electrolyte? Based on these two questions, is a paradigm change in the conventional understanding of the connection between the transport properties and cell performance needed? Furthermore, can the electrochemical stability of the individual electrolyte components be modified through coordination, and how does coordination affect interphase formation? These questions are addressed in the following sections, along with other aspects important for the future development, optimization, and implementation of highly concentrated electrolytes in lithium-based batteries.

## Comparison of bulk conductivity or lithium transference as a key transport property

The bulk electrolyte conductivity (the intrinsic ionic conductivity of a material, which is not affected by any interfaces, e.g., due to confinement in a porous structure) is often used as the key transport property to evaluate electrolytes prior to cell testing. Ionic conductivity is a parameter that can be screened relatively easily and reliably with the standard equipment available in most electrochemistry laboratories. Nonetheless, there are definite limitations; most notably, high bulk electrolyte conductivity does not necessarily imply a high lithium-ion conductivity^13^.

The contribution of the lithium-ion transport to the total current is known as the lithium transference number. The concept of a transference number is not unique to liquid lithium-ion electrolyte solutions. It is a general concept to describe the contribution of particular species *x* to the total (i.e., bulk) conductivity (transference number of *x*, T_*x*_, with values between 0 and 1)^14^. In “standard” electrolyte systems, this value is generally low (between 0.2 and 0.4)^15^, which means that the “free” anions are more mobile than the lithium ions with their solvent solvation shell. Thus, the anions, e.g. PF_6_^−^ a “standard” electrolyte as defined above, contribute more to the overall current. The lithium-ion conductivity, σ_Li_^+^, in a particular electrolyte can be determined from the product of the bulk electrolyte conductivity, σ, and T_Li_^+^ (i.e., σ_Li_^+^ = σ ∙ T_Li_^+^). Ultimately, it is the lithium-ion conductivity which limits the current density that can be achieved with a given electrolyte in an electrochemical cell^10^.

Research studies with highly-concentrated electrolytes have shown that electrolyte solutions with lower bulk conductivities can have improved electrochemical energy storage performances compared to their lower concentration counterparts^12,16^. This initially counterintuitive result implies that an electrolyte with lower bulk conductivity must have a higher lithium-ion conductivity, which can only be the case with higher T_Li_^+^ (i.e., if σ_Li_^+^[high] > σ_Li_^+^[low] as indicated by the cell performance and σ[high] < σ[low], then T_Li_^+^[high] > T_Li_^+^[low]). When the T_Li_^+^ values were given in peer-reviewed reports, they were higher in the highly-concentrated electrolytes than in more dilute “standard” electrolyte solutions (e.g., T_Li_^+^ = 0.42 for 1 M LiPF_6_ in EC/DMC (3:7 by vol) vs. T_Li_^+^ = 0.58 for 4 M LiTFSI + 0.5 M LiDFOB in fluoroethylene carbonate/DMC^16^ or T_Li_^+^ = 0.32 for 1 M LiPF_6_ in EC/DMC (1:2 by vol) vs. T_Li_^+^ = 0.57 for [LiFSI]:[acetonitrile]:[vinylene carbonate] = 0.52:1:0.09^17^).

A higher lithium-ion conductivity leads to higher availability of lithium ions at the electrode due to the formation of a lower concentration gradient within the electrolyte. A recent study using in situ Raman spectroscopy examined the amount of lithium ions in the electrolyte at a fixed position in the cell after the application of the current.^17^ The results showed a significant depletion of lithium ions in the “standard” carbonate electrolyte solution. However, the highly concentrated electrolyte solution (i.e., 10 M) formed a significantly lower concentration gradient. A further study has shown that although viscosity and ionic conductivity are good performance indicators of Li-ion cells with low mass loading (e.g., 0.95 mAh cm^−2^) and thin electrodes (e.g., 22 µm), higher concentration electrolytes (e.g., 1.9 M) could mitigate the depletion of lithium ions in the pores of thick electrodes (e.g., 8.10 mAh cm^−2^ mass loading and 161 µm thick) to improve electrochemical energy storage performance^18^. It is important to note that the ionic conductivity of the bulk electrolyte solution is generally higher than that of the electrolyte confined in the porous structure of the electrodes or separator^13^.

There would be advantages to using lithium-ion conductivity, derived from the transference number, as the key transport parameter to predict the behavior of electrolyte solutions. However, there are considerable challenges associated with accurately measuring the transference number. Significant variations have been reported by ref. 19 in the results obtained by electrochemical methods (e.g., the Bruce-Vincent method) and those coming from pulse-field gradient NMR^19^. Every method has particular restrictions/assumptions which have to be considered. Thus, transference numbers are not likely to be useful as a generalized screening tool. Nonetheless, the concept of transference and its impact on the lithium-ion concentration gradient in the battery cell should be taken into account in research works focusing on electrolytes.

## Impact of viscosity

**Fig. 2:**
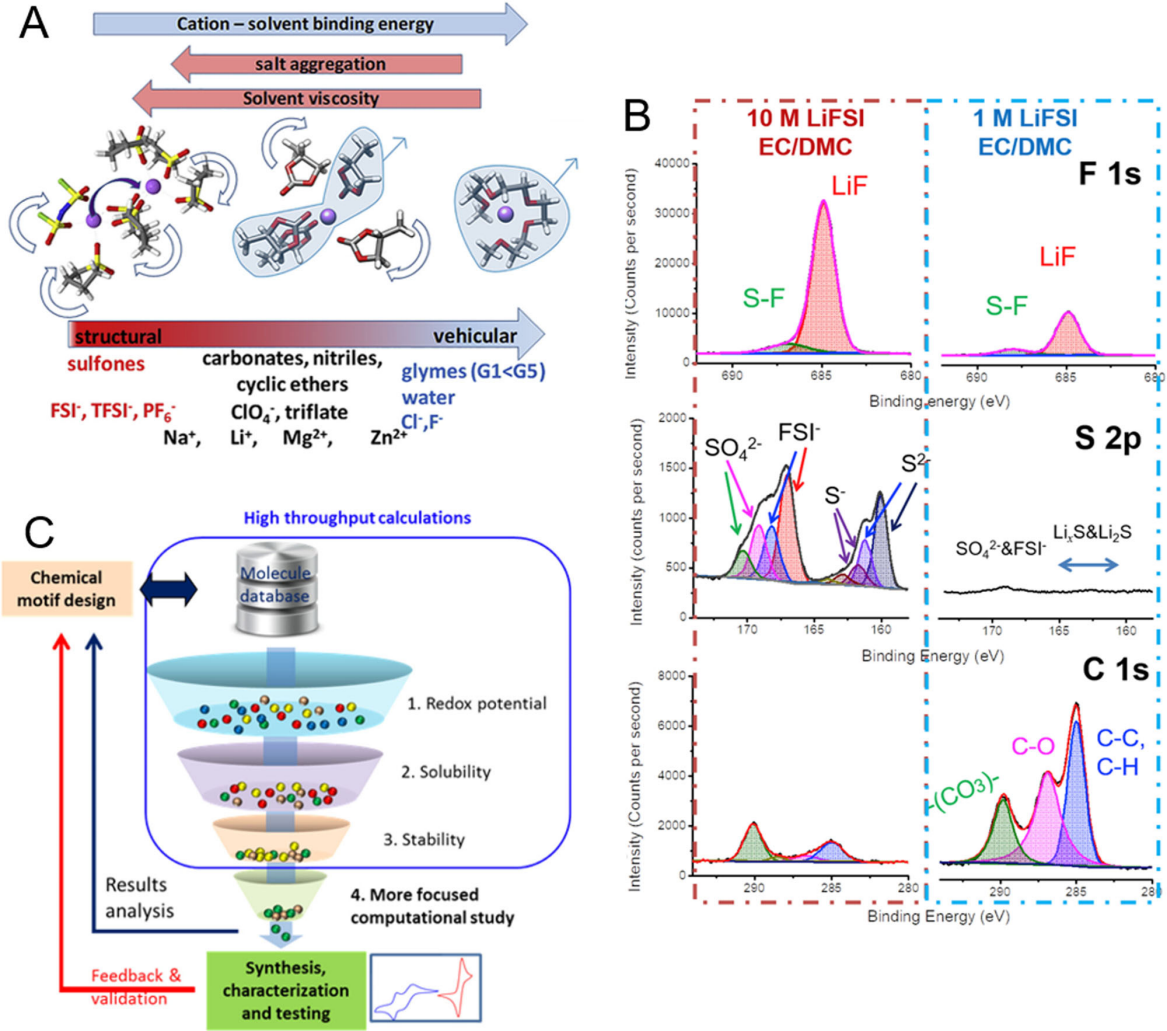
Aspects to consider for the development of highly-concentrated electrolyte solutions. **A** Parameters which influence the contribution of the vehicular and structural diffusion transport mechanisms for metal cations in liquid electrolytes (Reprinted from ref. 8 with permission from Elsevier.) **B** XPS analysis of Li metal cycled in concentrated (10 M) and standard (1 M) LiFSI in EC:DMC (1:1 by volume) electrolyte solution. (Reprinted from ref. 27 with permission from Elsevier). **C** Screening of material candidates using high-throughput calculations of key properties for focused computational studies and/or synthesis and testing. (Reproduced from Crabtree, G. *AIP Conference Proceedings* **1652**, 112–128 (2015)^36^, with the permission of AIP Publishing).

A high salt concentration in an electrolyte solution comes at the cost of high viscosity, which significantly hinders ion mobility. As noted above, the amount of solvent at high concentrations is no longer sufficient to completely fill the first solvation shell. Therefore, anions are involved in lithium-ion coordination. An insufficient amount of solvent can also lead to the scenario where the anions are coordinated to more than one lithium-ion to form what is often referred to as “aggregates”^8^. Aggregate formation along with anion coordination effectively increases the ionic radius of the solvated lithium ions. As mobility is inversely proportional to the product of the viscosity and the ion radius^14^, both of which are larger at high concentrations, the result is a significantly reduced mobility of the lithium-based complexes. This result is consistent with the classical picture of the forces (electric and drag) felt by a charged particle moving in an electrical field^14^ and describes the ion movement based on a vehicular mechanism of transport. However, studies have shown that when aggregates are present, the contribution of structural diffusion to the overall transport is significant (Fig. 2A)^8^, thus can help to negate the other effects of high viscosity^20^.

Electrolyte viscosity plays a role not only in the lithium-ion transport properties but also in aspects important to cell production and formation, namely electrolyte filling and wetting (although the wetting nature of the specific electrolyte, as defined by the contact angle, may play a more critical role)^21^. It has been shown, for example, that the capacity of lab-scale cells with high viscosity ionic liquid-based electrolyte solutions increases during initial cycling as the electrodes are progressively wetted with the electrolyte^20,22^. Various strategies could be used to understand and mitigate slow wetting. For example, solvents used as “diluents”, which do not change the local transport mechanism and solvation structure but reduce the viscosity, could be added to highly concentrated electrolyte solutions. This concept, which is relatively new and described as localized high-concentration electrolytes (LHCE)^23^, seems promising. In addition to modifying the electrolytes, analytical methods can be used to better understand and track wetting. For example, neutron radiography has been used to estimate the wetting degree^24^, while ultrasound propagation has been proposed as an in-line monitoring tool^25^.

## Different concentrations, different interphases

Beyond the transport properties, the electrochemical stability of the electrolyte and the interphase formation are critical aspects of obtaining satisfactory battery performance. In “standard” commercial electrolytes, the SEI/CEI formation is largely driven by the additives and electrolyte solvents, which are present in excess and largely uncoordinated by the lithium ions^4^. At high concentration, the electrochemical stability of the electrolyte and thus the interphases can be influenced by various factors, e.g., the presence of anions in the first solvation shell and lithium coordination of the majority of the solvent^26^. In this case, “high concentration” cannot be narrowed to a specific concentration range but should be defined based on coordination, as discussed above. A recent peer-reviewed article has given a detailed description of the complex interactions that take place between the cations, anions, and solvents in lithium-based non-aqueous electrolyte solutions^26^. An interesting result of several studies is the connection between anion coordination and the interphase composition^17,27^. The nature of the interphases is shifted from one largely dominated by the solvents and their decomposition products to one primarily influenced by the anions and their decomposition products, including LiF (Fig. 2B)^8,27^. Given the importance of the salt for the interphases with highly concentrated electrolytes, it is certainly interesting to explore other salts beyond LiPF_6_ and investigate the properties of the SEI/CEI that are formed. LiFSI has gained interest in this regard^17,27^. Other options include the use of dual salt systems^28^ or specific co-solvents (as in LHCEs)^23^. The approach of using mixtures of salts and/or solvents allows the advantages of the various electrolyte components to be exploited to address issues that can arise in other cell components (e.g., the corrosion of the Al current collector at the positive electrode)^16,23^.

## Development of electrolytes for next-generation lithium-based batteries

One of the factors driving research on highly concentrated electrolytes is the desire to enable cells capable of fully exploiting high-voltage cathode materials and lithium metal anodes^12,17,23^. High capacity or high voltage electrode materials are likely needed to compensate for the losses in energy content (particularly specific energy) due to the increased density of highly concentrated electrolytes. Even though high-concentration electrolytes have been used with graphite anodes (and generally paired with high-voltage cathodes), works focusing on a lithium metal electrode certainly dominate those reported in the literature^17,23,27,28^.

One of the major challenges with lithium metal anodes is the need to control the deposition morphology to avoid mossy or dendritic lithium metal growth. Highly-concentrated electrolyte solutions show a significant advantage over the “standard” electrolyte in this regard. Several studies^17,23^ have shown that highly-concentrated electrolytes (or also LHCEs) enable the deposition of lithium metal with a more dense and rounded deposited-lithium morphology at current densities of 1 mA cm^−2^. In similar conditions, needle-like dendritic structures are deposited from the “standard” 1 M carbonate-based electrolyte solutions^17,23^. In these examples, the authors attribute the improved lithium metal deposition (and dissolution) behavior to the changes in the composition of the SEI derived from concentrated electrolytes (where the SEI also depends on the composition of the electrolyte solution investigated)^17,23^.

In addition to the non-aqueous electrolytes that have been addressed here, other non-conventional electrolytes, such as water-in-salt electrolytes (WISE)^29^ or hybrid aqueous non-aqueous electrolytes (HANE)^30^, have been recently gaining interest. WISE and HANE take advantage of the complete solvent coordination (as in highly concentrated electrolytes as defined above) to extend the electrochemical stability window typical for aqueous electrolytes in other non-lithium-based energy storage technologies. However, significant research is still needed to allow these systems to compete with non-aqueous electrolytes for lithium-based batteries^29^.

Further development of novel electrolyte components and formulations can benefit from new research approaches involving high-throughput and autonomous testing platforms combined with machine learning. Computational screening of specific properties can limit the number of molecules subjected to in-depth studies (Fig. 2C)^31^. Autonomous platforms guided by machine-learning algorithms can be used to optimize formulations, possibly leading to non-intuitive electrolyte compositions with distinct properties, as has already been demonstrated with aqueous electrolyte solutions^32^. Furthermore, the use of advanced characterization techniques can lead to a better understanding of how the electrolyte behaves in the cell during operation. As an example, in situ Raman characterizations allowed lithium-ion depletion in the electrolyte to be directly investigated^18^. The combination of innovative research approaches with advanced analytical methods will most likely prove to be particularly important considering the multifaceted role of the electrolyte in a battery and its impact in terms of performance and lifetime.

The cost will ultimately be a driving factor in the implementation of high-concentration electrolytes. Although there are only relatively small differences between the costs of the main salts of interest, there is a factor of ~10 between the cost of the electrolyte solvents and the salts^33^. As a result, decreasing the amount of solvent and increasing the amount of salt leads to a net increase in the cost of the electrolyte formulation. As recently pointed out by the ref. 34, it is important to remember that the “costs” are not always monetary but can also be technical in nature, particularly when considering integrating new components into existing cell production processes.

The formation and aging step is the most costly step in terms of time and money in the production process of today’s Li-ion batteries^35^. Therefore, savings in this production step would be beneficial. Although it is known that the nature and composition of the interphases change in highly concentrated electrolytes, the potential impact (in terms of time and the associated monetary cost) on a formation step during cell production is still unknown. The question of cost is much more complex than that coming from the materials themselves. Thus, the benefits of the performance of highly-concentrated electrolyte solutions will likely have to be significant to outweigh the costs of the current “standard” 1 M electrolyte solutions.

For the foreseeable future, the “standard” 1 M electrolyte solutions are likely to remain state-of-the-art for the current generation of lithium-ion batteries. However, the encouraging experimental results obtained using highly concentrated electrolyte solutions could possibly open an alternative pathway toward future high-voltage and high-energy lithium-based batteries. Although bulk ionic conductivity will remain an accessible, reliable, and cost-effective screening tool for electrolyte development, it is important to remember that the highest bulk conductivity does not necessarily lead to the “best” cell performance, particularly in terms of rate capability. Leaving the conventional electrolyte wisdom behind to focus on aspects like the lithium-ion conductivity (i.e., transference) and the formation of concentration gradients within the electrolyte (leading to lithium-ion depletion) may inspire new avenues in electrolyte research using concentrations above the standard 1 M. Nonetheless, the importance of understanding interphase chemistry cannot be forgotten as the use of high-concentration electrolytes changes much of what scientists know about interphase formation.
